# Effect of antioxidants derived from Copaifera langsdorffii Desf. on skin tissue repair in diabetic rats

**DOI:** 10.1590/0034-7167-2025-0291

**Published:** 2026-07-31

**Authors:** Maria Neyze Martins Fernandes, Andreza Gysllaynny Delmondes Saraiva, Nathylle Régia de Sousa Caldas, Hercules Pereira Coelho, Maria do Socorro Vieira Lopes, Rita Neuma Dantas Cavalcante de Abreu, Woneska Rodrigues Pinheiro, Luis Rafael Leite Sampaio

**Affiliations:** I Universidade Regional do Cariri. Crato, Ceará, Brazil; II Universidade Estadual do Ceará. Fortaleza, Ceará, Brazil; III Universidade de Fortaleza. Fortaleza, Ceará, Brazil

**Keywords:** Diabetes *Mellitus*, Skin, Wound Healing, Antioxidants, Rats., Diabetes *Mellitus*, Piel, Cicatrización de Heridas, Antioxidantes, Ratas.

## Abstract

**Objectives:**

to assess the antioxidant effect of topically applied oleoresin from *Copaifera langsdorffii* Desf. on skin lesions in alloxan-induced diabetic rats.

**Methods:**

experiments were conducted on 200-300-gram adult male Wistar rats induced with diabetes by aloxan. A 7-millimeter metal punch was used for surgical circular epidermal excision. Animals were divided into five groups of five and treated with saline solution, mineral oil, 10% copaiba oil, essential fatty acids, or absolute copaiba oleoresin for one, seven, or 14 days. Malondialdehyde (MDA), nitrite, and glutathione (GSH) peroxidase concentrations were assessed to investigate oxidative activity progression. Tests were performed on animals approved by CEUA-URCA.

**Results:**

*Copaifera langsdorffii* Desf significantly reduced MDA and nitrite while increasing GSH. Groups treated with oleoresin, both 10% and absolute, improved assessed parameters.

**Conclusions:**

these results suggest that *Copaifera langsdorffii* Desf. oleoresin has antioxidant activity and may protect against oxidative stress in skin lesions of aloxan-induced diabetic rats.

## RESUMEN

**Objetivos:**

evaluar el efecto antioxidante de la aplicación de la oleorresina de *Copaifera langsdorffii* Desf. en lesiones cutáneas de ratas diabéticas inducidas por aloxano.

**Métodos:**

los experimentos se realizaron en ratas *Wistar* macho adultas entre 200 y 300 gramos. La escisión epidérmica se realizó utilizando un punch de 7 milímetros. Los animales se distribuyeron en cinco grupos (n=5), y se trataron con solución salina, aceite mineral, copaiba 10%, ácidos grasos esenciales o oleorresina de copaiba absoluta durante uno, siete o 14 días. Se evaluaron las concentraciones de malondialdehído (MDA), nitrito y glutatión (GSH) peroxidasa para investigar la progresión de la actividad oxidativa. El protocolo experimental fue aprobado por el CEUA-URCA.

**Resultados:**

*Copaifera langsdorffii* Desf. redujo significativamente los niveles de MDA y nitrito, mientras que aumentó la GSH. Los grupos tratados con oleorresina, tanto al 10% como en concentración absoluta, mostraron mejoría en los parámetros evaluados.

**Conclusiones:**

los resultados sugieren que la oleorresina de *Copaifera langsdorffii* Desf. presenta actividad antioxidante y puede proteger contra el estrés oxidativo en lesiones cutáneas de ratas diabéticas inducidas por aloxano.

## INTRODUCTION

Diabetes *mellitus* (DM) is a chronic disease that affects approximately 62 million people in the Americas and represents a significant challenge to global public health due to its various complications, such as retinopathy, peripheral neuropathy, and difficulties in tissue healing^(1,2)^. One of the most frequent and concerning complications is wound formation, characterized by delayed healing processes. The understanding of the process leading to the complexity of these wounds is not yet fully clear, but it is believed that oxidative stress plays a crucial role in lesion formation and evolution^(3)^. This delay in healing can be associated with chronic hyperglycemia, which increases free radical production, negatively affecting the growth and migration of cells responsible for tissue repair^(4,5)^.

Ulcers due to DM complications represent the most severe form of skin lesions and can lead to lower limb amputation^(6)^. Providing care to people with this type of wound is a daily concern and challenge for patients, caregivers, and healthcare professionals. In addition to causing discomfort and reducing quality of life, delayed wound healing also negatively impacts patient health and increases treatment costs^(7)^.

Although there are various treatment options for wounds, many are costly. Therefore, researchers and healthcare professionals seek new therapeutic approaches that are more economically accessible^(8)^. In this context, interest in research on topical therapies based on medicinal plants has increased. Meanwhile, *Copaifera* sp. oleoresin emerges as a promising alternative, both in its raw form and in gel formulations for local application. This is due to its beneficial properties, which include compounds such as α-copaene, trans-α-bergamotene, γ-muurolene, β-bisabolene, and sesquiterpenes α-humulene and β-caryophyllene^(9,10)^.

Copaiba cream at 10% was officially included in the Brazilian Pharmacopoeia Herbal Formulary as an anti-inflammatory and healing agent since 2011, mainly based on ethnobotanical studies^(11)^. However, in 2021, *Copaifera* spp. species were removed from this formulary^(12)^, due to a lack of robust and updated evidence on their efficacy. This change highlights the need for further research to validate and better understand copaiba’s therapeutic potential^(8)^.

Therefore, it is essential to explore more deeply copaiba’s therapeutic potential^(13)^, especially its antioxidant effect in treating ulcers due to DM complications. This study aims to assess the antioxidant effect of the topical application of raw oleoresin, comparing it with the 10% formulation and essential fatty acids (EFAs), recognized for its antioxidant properties^(14)^.

## OBJECTIVES

To assess the antioxidant effect of topically applied oleoresin from *Copaifera langsdorffii* Desf. on skin lesions in alloxan-induced diabetic rats.

## METHODS

### Ethical and legal considerations

The entire research proposal is in compliance and was conducted in strict adherence to current bioethical standards and guidelines: non-human animals (Guide for the care and use of laboratory animals, National Institute of Health, United States of America (USA), 1996; Federal Law 11,794/2008; Ethical Principles of Animal Experimentation from the Brazilian College of Animal Experimentation).

The experiments involving animals followed the experimental protocols approved in advance by the Ethics Committee on Animal Use at the *Universidade Regional do Cariri* (URCA). The entire study complied with the principles of Good Laboratory Practices and Good Manufacturing Practices and Control for the safety of researchers.

### Study design, location, and period

This is an experimental study, developed in accordance with the of Animal Research: Reporting *In Vivo* Experiments (ARRIVE) guidelines^(15)^, conducted from January to May 2022 at the Laboratory of Pharmacological Technologies and Innovations at URCA in Ceará, Brazil.

### Population and sample

Adult male Wistar albino rats, weighing between 200 and 300 grams (g), were used in this study. The animals were obtained from the Animal House at the URCA and were kept in a room with controlled conditions, with temperature maintained at 23 ± 1°C, a 12-hour light/dark cycle, and free access to food and water. Relative humidity was controlled, averaging between 40% and 70%, in accordance with the guidelines established by the Brazilian National Council for the Control of Animal Experimentation (In Portuguese, *Conselho Nacional de Controle de Experimentação Animal* - CONCEA).

The animals were randomly allocated into five groups, each comprising five rats: saline solution (SS), mineral oil (MO), 10% copaiba oil (10% CO), EFA, and absolute copaiba oil (CO). Each group was further subdivided into three subgroups, observed at different time points (one, seven, and 14 days), totaling 75 animals used in the study. A sample size calculation was performed to ensure the fewest possible animals were used while respecting the 3Rs principle and guaranteeing statistically robust and ethically responsible results. A significance level (α) of 5% and a statistical power of 80% were adopted. With these parameters, the minimum estimated sample size was 15 animals per group, for a total of 75 animals across the five experimental groups. This number allows detection of statistically significant differences among groups, balancing scientific rigor and reduction in animal use in accordance with CONCEA and ARRIVE.

### Study protocol - anesthesia

After recording the animals’ body weights, the rats were anesthetized intraperitoneally with xylazine (10 mg/kg) and ketamine (100 mg/kg)^(16)^.

### Study protocol - experimental diabetes induction

To induce diabetes, the animals were subjected to a 24-hour fast. After this period, they were anesthetized with xylazine at 10 mg/kg and ketamine at 100 mg/kg for administering alloxan at a dose of 50 mg/kg via the dorsal penile vein. Six hours after alloxan injection, a 10% glucose solution was provided for a 24-hour period. To verify diabetes, blood glucose levels were checked 72 hours after alloxan administration and on the day of euthanasia. Animals that did not show values equal to or greater than 250 milligrams (mg) of glucose per deciliter of blood were discarded^(17)^. The checks were performed by taking a blood sample from the anesthetized animal’s tail, dropping the sample onto Accu-Chek Active^®^ test strips, and reading the results on an Accu-Chek Active^®^ device.

### Study protocol - shaving and cutaneous wound production

After confirming diabetes and anesthesia, the animals were placed in a prone position, and their dorsal area was manually shaved. The surgical field was then aseptically prepared with povidone-iodine, and a surgical excision of a skin fragment, measuring approximately 7 millimeters (mm) in total length and about 2 mm in depth, was performed using a punch. Excision was standardized to ensure that all layers were properly removed, except for the underlying musculature^(18)^. Subsequently, the wounds were measured, analyzed, and the animals were individually returned to their respective cages.

### Study protocol - treatment protocol

**Figure 1:**
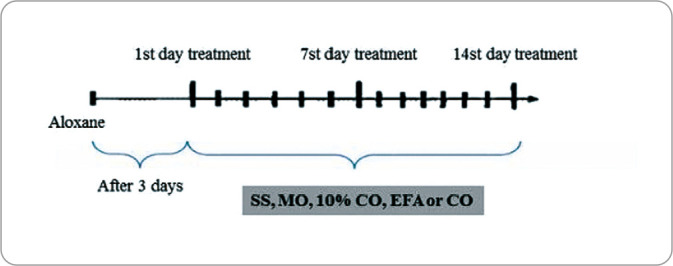
Treatment protocol with saline solution (SS), mineral oil (MO), 10% copaiba oil (CO10%), essential fatty acid (EFA), and absolute copaiba oil (CO)

The animals were divided into five groups, each composed of 15 animals, to receive a daily topical application of 0.2 milliliter of the corresponding treatment compound (Figure 1) for observation periods of one, seven, and 14 days^(19)^.

### Study protocol - copaiba oil extraction

To obtain the oil, a formal request was made through the *Sistema de Autorização e Informação em Biodiversidade*, regulated by Normative Instruction 03/2014, which governs the collection of biological material for scientific purposes and research. Additionally, authorization was obtained for the extraction of CO from *Instituto Chico Mendes de Conservação da Biodiversidade* in Crato, Ceará, located at the following address: R. Dr. Quixadá Felício, S/N - Pimenta, Crato - CE, 63105-030.

After obtaining consent, extraction was carried out in the Araripe National Forest, located in the municipality of Crato, Ceará, Brazil. The forest is at an altitude of 909 meters above sea level and has coordinates of 7°34’05.26”S and 39°43’54.00”W. A GPS device provided the location information. To ensure identification^(20)^, a representative sample of the species was collected and deposited at the Dárdano de Andrade e Lima Herbarium (part of URCA) as an exsiccate (specimen number 14208).

The oil was collected by randomly drilling the tree trunk at two different locations using a conventional auger with a diameter of 2 centimeters (cm) and a length of 45 cm, with two openings at heights of 1 meter (m) and 1.50 m. After the required amount of oil had drained, the hole was sealed with a 3/4-inch-diameter polyvinyl chloride pipe that was 10 cm long and capped with a plastic lid. To facilitate future collections and avoid wood residue buildup, the pipes or hoses could be removed after collection was complete and wooden threads could be attached to the holes to prevent pests from entering^(21)^. The collected material was stored in a light-proof container and kept in the refrigerator to prevent material oxidation.

The 10% CO formulation was obtained using MO as a diluent. MO rarely causes allergic reactions, does not solidify or clog pores, and is used to dilute essential oils in pharmacological practice^(22)^.

### Analysis of results - antioxidant effect

At the end of the treatment period, the animals were dissected to prepare a 10% (w/v) homogenate in a 0.05 M phosphate buffer at pH 7.4. Then, the animals were sacrificed to analyze the antioxidant effect.

Measurement of lipid peroxidation from tissue homogenate samples obtained from the animals’ skin was analyzed. The samples were added to a free radical formation catalyst system (FeSO_4_ 0.01 mm and ascorbic acid 0.1 mm) and maintained at 37°C for 30 minutes. The reaction was stopped by adding 10% trichloroacetic acid. Then, the samples were centrifuged at 3,000 rpm for 15 minutes. The supernatant was removed and added to 0.8% thiobarbituric acid. The mixture was placed in a water bath for 15 minutes. After cooling, absorbance was measured at 535 nm. Lipid peroxidation was expressed in µmol of malondialdehyde (MDA) per mg of tissue^(23)^.

The nitrite concentration in the rat skin homogenate was determined immediately after dissection of all groups. After centrifugation at 800×g for ten minutes, the supernatant was collected from the homogenate, and nitric oxide (NO) production was determined based on the Griess reaction^(24)^. To do so, 100µL of the supernatant was incubated with 100µL of the Griess reagent (sulfanilamide in 1% H3PO4/0.1% N-(1-naphthyl)ethylenediamine-dihydrochloride/1% H_3_PO_4_/distilled water, 1:1:1:1) at room temperature for ten minutes. Absorbance was measured at 550 nm using a microplate reader. A standard curve was created using various concentrations of NaNO₂ ranging from 0.75 to 100 µM, and the results were expressed in µmol/g of protein.

To determine reduced glutathione (GSH) concentration, 10% (w/v) homogenates in 0.02 M EDTA were added to a 50% trichloroacetic acid solution. After centrifugation at 3,000 rpm for 15 minutes, the homogenized supernatant was collected, and GSH production concentration was determined^(25)^. In summary, the samples were mixed with 0.4 M Tris-HCl buffer, pH 8.9, and 0.01 M DTNB. The GSH level was determined by absorbance at 412 nm and expressed as ng/g of wet tissue weight of GSH.

### Statistical analysis

All analyses were performed using analysis of variance (ANOVA) with the Prism 8.0.2 software for Windows from GraphPad software (San Diego, CA, USA). Multiple comparisons for significance were conducted with ANOVA and Tukey post-hoc tests. Results are considered significant at p < 0.05 and presented as mean ± Standard Error of the Mean^(26)^.

## RESULTS

Moreover, 10% CO, EFA, and CO administration on MDA concentration in diabetic rat wounds induced by alloxan and subjected to different administration regimens over one, seven, or 14 days are evident.

**Figure 2:**
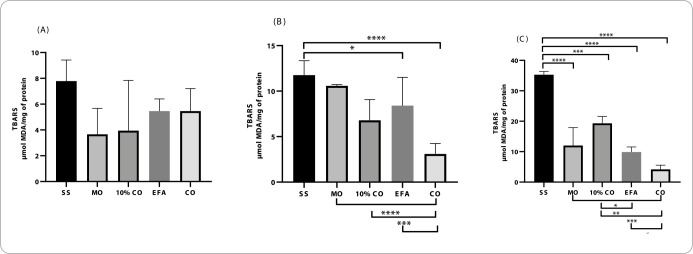
Effects of copaiba oil on malondialdehyde concentration in wounds in alloxan-induced diabetic rats. Each bar represents the mean ± Standard Error of the Mean of groups treated with saline solution (SS), mineral oil (MO), 10% copaiba oil (10% CO), essential fatty acid (EFA), and copaiba oil (CO)

Regarding acute treatment with these substances, no significant effects were observed in MDA concentrations when compared to SS (Figure 2A). However, when repeated doses were administered over seven days, it was found that both CO (3.0 ± 0.6) and EFA (8.4 ± 1.5) induced a significant reduction in MDA concentrations compared to SS (11.7 ± 0.9). Additionally, in comparison to the CO group, there was a decrease compared to MO (10.5 ± 0.0), 10% CO (6.7 ± 1.6), and EFA groups (Figure 2B).

*(A) Animals treated for one day; (B) Animals treated with repeated doses for seven days; (C) Animals treated with repeated doses for 14 days. For all analyses, p < 0.05 was considered significant (analysis of variance followed by Tukey’s post-hoc test).*

In the scenario of repeated treatment over 14 days, MO (12.0 ± 4.1), 10% CO (19.3 ± 1.6), EFA (9.8 ± 0.8) and CO (4.1 ± 0.6) groups demonstrated considerable reductions in MDA quantities when compared to SS (35.2 ± 0.6). Furthermore, this decrease was equally notable in the results of the EFA group compared to the 10% CO group, and in the CO group compared to the MO, 10% CO, and EFA groups (Figure 2C).

### Nitrite determination

Three graphical representations were constructed to illustrate nitrite analysis results, delineating the effects observed concerning different treatment periods and applied substances.

**Figure 3:**
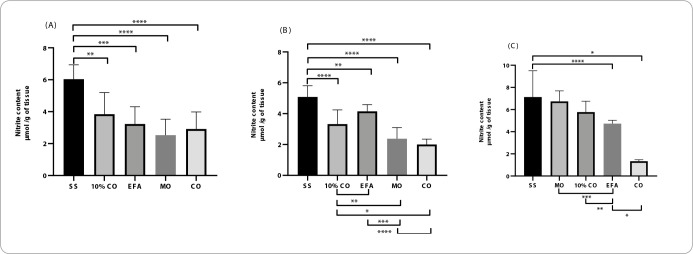
Effects of copaiba oil on nitrite concentration in lesions of aloxan-induced diabetic rats. Each bar represents the mean ± Standard Error of the Mean of the groups treated with saline solution (SS), mineral oil (MO), 10% copaiba oil (10% CO), essential fatty acid (EFA), and copaiba oil (CO)

In the acute administration of topical treatment, a reduction in nitrite concentration was observed when using MO (3.8 ± 0.6), 10% CO (3.2 ± 0.4), EFA (2.5 ± 0.4), and CO (2.9 ± 0.4), compared to SS (6.0 ± 0.4) (Figure 3A).

*(A) Animals treated for one day; (B) Animals treated with repeated doses for seven days; (C) Animals treated with repeated doses for 14 days. For all analyses, p < 0.05 was considered significant (analysis of variance followed by Tukey’s post-hoc test).*

Similar results related to nitrite concentration, as described above, were evident in groups treated with MO (3.3 ± 0.4), 10% CO (4.1 ± 0.2), EFA (2.3 ± 0.3) and CO (2.0 ± 0.1) through repeated doses for seven days, compared to SS (5.0 ± 0.3) (Figure 3B). It is worth noting that MO showed a lower nitrite concentration compared to the 10% CO group, while EFA exhibited a lower concentration when compared to MO and 10% CO. On the other hand, treatment with CO showed a reduction when compared to MO, 10% CO, and EFA.

Throughout the treatment repeated for 14 days, both EFA (4.7 ± 0.1) and CO (1.3 ± 0.0) exhibited a decrease in nitrite concentration compared to SS (7.1 ± 1.0) (Figure 3C). The EFA group also showed a reduction when contrasted with the MO (6.7 ± 0.4) and 10% CO (5.7 ± 0.4) groups. Furthermore, treatment with CO showed greater significance in reducing nitrite concentration compared to the MO, 10% CO, and EFA groups.

### Glutathione concentration determination

**Figure 4:**
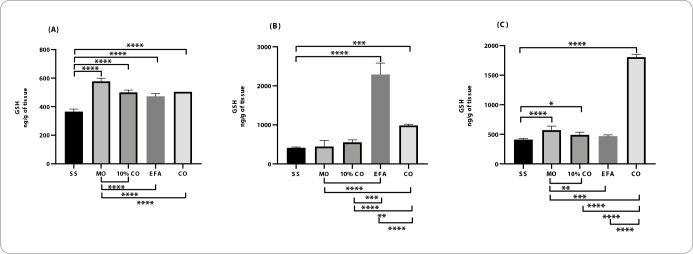
Effects of copaiba oil on glutathione concentration in skin lesions in aloxan-induced diabetic rats. Each bar represents the mean ± Standard Error of the Mean of groups treated with saline solution (SS), mineral oil (MO), 10% copaiba oil (10% CO), essential fatty acid (EFA), and copaiba oil (CO)

In the acute treatment phase, MO (577.7 ± 9.8), 10% CO (500.7 ± 7.6), EFA (472.7 ± 9.3), and CO (504.2 ± 0.0) showed an increase in GSH concentration compared to SS (365.9 ± 10.2). When comparing the effects of MO treatment to the 10% CO, EFA, and CO groups after one day of application, it was found that MO resulted in a significant increase in GSH concentration (Figure 4A).

*(A) Animals treated for one day; (B) Animals treated repeatedly for seven days; (C) Animals treated repeatedly for 14 days. For all analyses, p < 0.05 was considered significant (analysis of variance followed by Tukey’s post-hoc test).*

In the treatment with repeated doses over seven days, it was evident that the EFA (2290 ± 169.1) and CO (986.7 ± 17.2) groups showed an increase in GSH concentration compared to SS (414.8 ± 7.9) (Figure 4B). Additionally, over these seven days, treatment with EFA showed a higher increase when compared to the groups that received MO (445.5 ± 69.8), 10% CO (552.6 ± 0.0), and CO groups. Similarly, the CO group had a higher GSH concentration compared to the MO and 10% CO groups.

In the context of treatment lasting 14 days, the performance of the MO (571.7 ± 33.7), 10% CO (490.2 ± 22.8), and CO (1805.0 ± 34.9) groups is highlighted, all of which showed a significant increase in GSH concentration compared to SS (411.1 ± 9.1) (Figure 4C). These positive results of CO treatment remained consistent when compared to the effects of MO, 10% CO, and EFA (471.6 ± 9.5), maintaining elevated GSH concentrations. Additionally, MO increased GSH concentration compared to 10% CO and EFA.

## DISCUSSION

The analyses demonstrated that absolute CO showed superior results in reducing MDA concentration compared to EFA and the other treatment groups (SS, MO, and 10% CO).

In this context, the reduction in MDA concentration observed in the group treated with absolute copaiba oleoresin indicates decreased lipid peroxidation, a process that can be critical during the inflammatory and proliferative phases of wound healing. MDA is the final product of lipid peroxidation^(23)^, and reducing it suggests effective control of oxidative stress, which may favor the resolution of inflammation and the transition to the proliferative phase. This could potentially reduce oxidative attacks on cellular components that are repaired during the healing process. Such damage is frequently observed in ulcerations resulting from DM complications without antioxidant treatment^(4,5)^. Similar^(18,27)^ results demonstrated that reducing MDA levels is directly associated with improved inflammatory parameters and accelerated wound healing in diabetic rats treated with alpha-lipoic acid, highlighting oxidative stress modulation as a key factor for the proper progression of tissue repair phases.

This potential anti-inflammatory activity may be attributed to the presence of flavonoids and kaurenoic acid^(28)^ in absolute copaiba oleoresin, which contribute to free radical scavenging and lipid oxidation inhibition, thereby preventing damage to cell membranes^(29)^. Studies^(30,31)^ indicate that these bioactive compounds act by modulating antioxidant enzymes and decreasing the activity of myeloperoxidase, an enzyme involved in the generation of Reactive Oxygen Species (ROS) during the inflammatory phase.

A significant increase in MDA concentration was observed in the SS group on day 14, which may be attributed to the temporal progression of oxidative damage to cellular membranes in lesions of diabetic rats treated with a formulation lacking antioxidant agents. DM is associated with increased lipid peroxidation, resulting in elevated MDA levels, a recognized marker of oxidative stress. This process is linked to the chronic hyperglycemia characteristic of DM, which promotes excessive ROS production, leading to oxidative damage to lipids, proteins, and DNA, along with a reduction in endogenous antioxidant defenses^(32)^.

Regarding nitrite concentration modulation, it was observed that after seven days of treatment, only EFA and CO maintained effective biological activity in reducing this marker, suggesting that the other formulations are more effective during the acute phase of the repair process. EFA showed greater antioxidant activity compared to 10% CO and MO. However, CO exhibited superior performance in nitrite reduction, indicating a more sustained and consistent effect on oxidative stress modulation. These findings reinforce that absolute copaiba oleoresin exerts a prolonged therapeutic effect with the capacity to maintain redox homeostasis over time.

This effect may be associated with CO’s ability to neutralize ROS, particularly hydrogen peroxide (H₂O₂)^(33)^, which is closely related to its efficacy in modulating oxidative stress throughout the different phases of wound healing in DM. Moreover, in the context of DM, H₂O₂ can react with NO to form peroxynitrite radicals^(34)^, highly reactive compounds that cause significant cellular damage^(35)^. This damage can impair the early stages of wound healing, such as the inflammatory phase and granulation tissue formation, ultimately delaying the tissue repair process. Similar results were also reported by^(18,27)^, who found that reducing NO in diabetic wounds leads to lower formation of nitrogen-derived radicals, promoting reduced inflammation and better granulation tissue organization in subsequent healing phases.

Therefore, the topical application of CO may represent a promising therapeutic approach to enhance wound healing in individuals with DM by neutralizing ROS and promoting a redox environment conducive to tissue regeneration.

Another key enzyme in the defense against oxidative stress is GSH, an endogenous antioxidant that plays a crucial role in maintaining cellular homeostasis^(36)^. This study observed that during the initial inflammatory phase of wound healing, particularly compromised in DM due to elevated oxidative stress, there was an increase in GSH concentrations in all treated groups. However, EFA exhibited a peak effect on day seven, followed by a decline on day 14, indicating that its antioxidant effect is limited to the early stages of healing.

In contrast, CO demonstrated a superior ability to sustain GSH concentrations over time, including during the proliferative phase and the early stages of tissue remodeling, compared to other treatments (SS, MO, and 10% CO). This effect suggests that CO exerts a more prolonged antioxidant effect, which is particularly relevant in the context of DM, where wound healing is often impaired by persistent oxidative stress.

CO’s effect may be partly attributed to the presence of kaurenoic acid, whose ability to stimulate GSH synthesis or preserve its levels has already been demonstrated^(37)^. This compound plays a fundamental role in enhancing endogenous antioxidant defenses, particularly in hyperglycemic conditions like those seen in DM. It contributes to a more favorable biochemical microenvironment for wound healing and supports the efficient progression of repair phases. Similar findings^(18,27)^ indicate that elevated GSH levels are crucial for accelerating the proliferative phase and promoting proper extracellular matrix deposition and re-epithelialization in diabetic skin wounds.

On the other hand, MO and 10% CO did not show significant efficacy in maintaining GSH levels, presenting inferior responses compared to CO and EFA. This finding is supported by^(38)^, which reports that low CO concentrations do not exhibit robust antioxidant activity, explaining the limited efficacy observed in the 10% formulation used in this study.

Based on the results, it is concluded that *Copaifera langsdorffii* Desf. demonstrates a relevant antioxidant effect in the wound healing process of animals with induced DM, acting to reduce MDA and nitrite concentrations while promoting an increase in GSH. CO’s superior performance, particularly during the inflammatory and proliferative phases of healing, highlights its therapeutic potential in mitigating oxidative stress, one of the main factors impairing tissue repair in DM. Nonetheless, further research is needed to elucidate its molecular mechanisms and to establish standardized clinical protocols to ensure its safe and effective application.

### Study limitations

This research was characterized as a pre-clinical study to elucidate the antioxidant role of *Copaifera langsdorffii* Desf. in diabetes-related ulcer healing. Clinical studies are needed to incorporate antioxidants such as *Copaifera langsdorffii* Desf. into healthcare practice.

### Contributions to health, nursing or public policy

The search for therapies that minimize oxidative damage in diabetic ulceration has been frequent in wound care units. In this context, as a contribution to the advancement in the practice of care and nursing science, this study presents important results from a natural resource that expresses scientific evidence and can be used in nursing care for treating diabetes-related ulcers.

## CONCLUSIONS

Copaiba oleoresin demonstrated positive effects in reducing MDA concentrations after repeated doses for seven or 14 days of treatment, lowered nitrite levels at all treatment periods, and increased GSH’s potential after repeated doses for seven and 14 days.

In conclusion, *Copaifera langsdorffii* Desf. oleoresin is a potential therapeutic agent for the topical treatment of MDA, nitrite, and GSH parameters induced by aloxan in rat lesions.

## Data Availability

The research data are available only upon request.
