# Supplementary material for: Effect of antioxidants derived from Copaifera langsdorffii Desf. on skin tissue repair in diabetic rats
Source: Rev Bras Enferm. 2026 Jul 31;79:e20250291. doi: 10.1590/0034-7167-2025-0291 (PMC13435860; doi:10.1590/0034-7167-2025-0291)
Supplement: Translation

# Translation: Efeito de antioxidantes derivados de Copaifera langsdorffii Desf. na reparação tecidual cutânea em ratos diabéticos

**DOI:** 10.1590/0034-7167-2025-0291pt

**Authors:** Maria Neyze Martins Fernandes, Andreza Gysllaynny Delmondes Saraiva, Nathylle Régia de Sousa Caldas, Hercules Pereira Coelho, Maria do Socorro Vieira Lopes, Rita Neuma Dantas Cavalcante de Abreu, Woneska Rodrigues Pinheiro, Luis Rafael Leite Sampaio

**Affiliations:** I Universidade Regional do Cariri. Crato, Ceará, Brasil; II Universidade Estadual do Ceará. Fortaleza, Ceará, Brasil; III Universidade de Fortaleza. Fortaleza, Ceará, Brasil

**Keywords:** Diabetes *Mellitus*, Pele, Cicatrização, Antioxidantes, Ratos.

## Abstract

**Objetivos:**

avaliar efeito antioxidante da aplicação de óleo-resina de *Copaifera langsdorffii* Desf. em lesões cutâneas de ratos diabéticos induzidos por aloxano.

**Métodos:**

conduziram-se experimentos em ratos *Wistar* machos adultos com 200 a 300 gramas. Realizou-se excisão epidérmica com *punch* de 7 milímetros. Animais foram divididos em cinco grupos (n=5) e tratados com solução salina, óleo mineral, óleo de copaíba 10%, ácidos graxos essenciais ou óleo-resina de copaíba absoluta por um, sete ou 14 dias. Concentrações de malondialdeído (MDA), nitrito e glutationa (GSH) peroxidase foram avaliadas para investigar progressão da atividade oxidativa. Protocolo experimental foi aprovado pelo CEUA-URCA.

**Resultados:**

*Copaifera langsdorffii* Desf. reduziu significativamente MDA e nitrito, enquanto aumentou GSH. Grupos tratados com óleo-resina, tanto a 10% quanto em concentração absoluta, apresentaram melhora nos parâmetros avaliados.

**Conclusões:**

resultados sugerem que óleo-resina de *Copaifera langsdorffii* Desf., possui atividade antioxidante, podendo proteger contra estresse oxidativo em lesões cutâneas de ratos diabéticos por aloxano.

## INTRODUÇÃO

O diabetes *mellitus* (DM) é uma doença crônica que afeta aproximadamente 62 milhões de pessoas nas Américas e representa um desafio significativo para a saúde pública global devido às suas diversas complicações, como retinopatia, neuropatia periférica e dificuldades na cicatrização de tecidos^(1,2)^. Uma das complicações mais frequentes e preocupantes é a formação de feridas, caracterizada por processos de cicatrização retardados. A compreensão do processo que leva à complexidade dessas feridas ainda não está totalmente esclarecida, mas acredita-se que o estresse oxidativo desempenhe um papel crucial na formação e evolução da lesão^(3)^. Esse atraso na cicatrização pode estar associado à hiperglicemia crônica, que aumenta a produção de radicais livres, afetando negativamente o crescimento e a migração das células responsáveis pelo reparo tecidual^(4,5)^.

Úlceras decorrentes de complicações do DM representam a forma mais grave de lesões cutâneas e podem levar à amputação de membros inferiores^(6)^. Cuidar de pessoas com esse tipo de ferida é uma preocupação e um desafio diários para pacientes, cuidadores e profissionais de saúde. Além de causar desconforto e reduzir a qualidade de vida, a cicatrização tardia da ferida também impacta negativamente a saúde do paciente e aumenta os custos do tratamento^(7)^.

Embora existam diversas opções de tratamento para feridas, muitas são dispendiosas. Portanto, pesquisadores e profissionais de saúde buscam novas abordagens terapêuticas que sejam mais acessíveis economicamente^(8)^. Nesse contexto, o interesse em pesquisas sobre terapias tópicas à base de plantas medicinais tem aumentado. Enquanto isso, a oleorresina de *Copaifera* sp. surge como uma alternativa promissora, tanto em sua forma bruta quanto em formulações em gel para aplicação local. Isso se deve às suas propriedades benéficas, que incluem compostos como α-copaeno, trans-α-bergamoteno, γ-muuroleno, β-bisaboleno e os sesquiterpenos α-humuleno e β-cariofileno^(9,10)^.

O creme de copaíba a 10% foi oficialmente incluído no Formulário de Fitoterápicos da Farmacopeia Brasileira como agente anti-inflamatório e cicatrizante desde 2011, com base principalmente em estudos etnobotânicos^(11)^. No entanto, em 2021, as espécies de *Copaifera* spp. foram removidas deste formulário^(12)^, devido à falta de evidências robustas e atualizadas sobre sua eficácia. Essa mudança destaca a necessidade de mais pesquisas para validar e compreender melhor o potencial terapêutico da copaíba^(8)^.

Portanto, é essencial explorar mais profundamente o potencial terapêutico da copaíba^(13)^, especialmente seu efeito antioxidante, no tratamento de úlceras decorrentes de complicações do DM. Este estudo visa avaliar o efeito antioxidante da aplicação tópica da oleorresina bruta, comparando-a com a formulação a 10% e com ácidos graxos essenciais (AGE), reconhecidos por suas propriedades antioxidantes^(14)^.

## OBJETIVOS

Avaliar o efeito antioxidante da oleorresina de *Copaifera langsdorffii* Desf. aplicada topicamente em lesões cutâneas de ratos diabéticos induzidos por aloxano.

## MÉTODOS

### Considerações éticas e legais

Toda a proposta de pesquisa está em conformidade e foi conduzida em estrita observância às normas e diretrizes bioéticas vigentes: animais não humanos (Guia para o cuidado e uso de animais de laboratório, *National Institute of Health*, Estados Unidos da América (EUA), 1996; Lei Federal n^o^ 11.794/2008; Princípios Éticos da Experimentação Animal do Colégio Brasileiro de Experimentação Animal).

Os experimentos envolvendo animais seguiram os protocolos experimentais previamente aprovados pelo Comitê de Ética no Uso de Animais da Universidade Regional do Cariri (URCA). Todo o estudo cumpriu os princípios das Boas Práticas de Laboratório e das Boas Práticas de Fabricação e Controle para a segurança dos pesquisadores.

### Desenho, localização e período do estudo

Trata-se de estudo experimental, desenvolvido de acordo com as diretrizes do *Animal Research: Reporting In Vivo Experiments* (ARRIVE)^(15)^, conduzido de janeiro a maio de 2022 no Laboratório de Tecnologias e Inovações Farmacológicas da URCA no Ceará, Brasil.

### População e amostra

Ratos *Wistar* albinos machos adultos, pesando entre 200 e 300 gramas (g), foram utilizados neste estudo. Os animais foram obtidos do Biotério da URCA e mantidos em uma sala com condições controladas, com temperatura mantida a 23±1°C, ciclo claro/escuro de 12 horas e livre acesso a água e ração. A umidade relativa foi controlada, variando em média entre 40% e 70%, de acordo com as diretrizes estabelecidas pelo Conselho Nacional de Controle de Experimentação Animal (CONCEA).

Os animais foram distribuídos aleatoriamente em cinco grupos, cada um composto por cinco ratos: solução salina (SS), óleo mineral (OM), óleo de copaíba a 10% (OC 10%), AGE e óleo de copaíba (OC) puro. Cada grupo foi subdividido em três subgrupos, observados em diferentes momentos (um, sete e 14 dias), totalizando 75 animais utilizados no estudo. Um cálculo do tamanho da amostra foi realizado para garantir o uso do menor número possível de animais, respeitando o princípio dos 3Rs e assegurando resultados estatisticamente robustos e eticamente responsáveis. Um nível de significância (α) de 5% e um poder estatístico de 80% foram adotados. Com esses parâmetros, o tamanho mínimo estimado da amostra foi de 15 animais por grupo, totalizando 75 animais nos cinco grupos experimentais. Esse número permite a detecção de diferenças estatisticamente significativas entre os grupos, equilibrando o rigor científico e a redução do uso de animais, em conformidade com os princípios CONCEA e ARRIVE.

### Protocolo de estudo - anestesia

Após o registro do peso corporal dos animais, os ratos foram anestesiados por via intraperitoneal com xilazina (10 mg/kg) e cetamina (100 mg/kg)^(16)^.

### Protocolo de estudo - indução experimental de diabetes

Para induzir diabetes, os animais foram submetidos a um jejum de 24 horas. Após esse período, foram anestesiados com xilazina (10 mg/kg) e cetamina (100 mg/kg) para a administração de aloxano (50 mg/kg) por via intravenosa na veia dorsal do pênis. Seis horas após a injeção de aloxano, foi administrada uma solução de glicose a 10% por um período de 24 horas. Para confirmar o diagnóstico de diabetes, os níveis de glicose no sangue foram verificados 72 horas após a administração de aloxano e no dia da eutanásia. Os animais que não apresentaram valores iguais ou superiores a 250 miligramas (mg) de glicose por decilitro de sangue foram descartados^(17)^. Os testes foram realizados coletando uma amostra de sangue da cauda do animal anestesiado, depositando a amostra em tiras de teste *Accu-Chek Active* ^®^ e lendo os resultados em um dispositivo *Accu-Chek Active* ^®^.

### Protocolo de estudo - raspagem e produção de feridas cutâneas

Após a confirmação do diabetes e da anestesia, os animais foram posicionados em decúbito ventral, e a região dorsal foi tricotomizada manualmente. O campo cirúrgico foi então preparado assepticamente com iodopovidona, e um fragmento de pele, medindo aproximadamente 7 milímetros (mm) de comprimento total e cerca de 2 mm de profundidade, foi removido cirurgicamente com um *punch*. A excisão foi padronizada para garantir a remoção completa de todas as camadas, exceto a musculatura subjacente^(18)^. Em seguida, as feridas foram medidas e analisadas, e os animais foram devolvidos individualmente às suas respectivas gaiolas.

### Protocolo de estudo - protocolo de tratamento

Os animais foram divididos em cinco grupos, cada um composto por 15 animais, para receber uma aplicação tópica diária de 0,2 mililitros do composto de tratamento correspondente (Figura 1) durante períodos de observação de um, sete e 14 dias^(19)^.

**Figura 1:** Protocolo de tratamento com solução salina (SS), óleo mineral (OM), óleo de copaíba a 10% (OC10%), ácido graxo essencial (AGE) e óleo de copaíba (OC) absoluto

### Protocolo de estudo - extração de óleo de copaíba

Para obtenção do óleo, foi feita solicitação formal por meio do Sistema de Autorização e Informação em Biodiversidade, regulamentado pela Instrução Normativa n^o^ 03/2014, que disciplina a coleta de material biológico para fins científicos e de pesquisa. Adicionalmente, foi obtida autorização para extração de OC do Instituto Chico Mendes de Conservação da Biodiversidade em Crato, Ceará, localizado no seguinte endereço: R. Dr. Quixadá Felício, S/N - Pimenta, Crato - CE, 63105-030.

Após a obtenção do consentimento, a extração foi realizada na Floresta Nacional do Araripe, localizada no município de Crato, Ceará, Brasil. A floresta está a uma altitude de 909 metros acima do nível do mar e possui coordenadas de 7°34’05.26”S e 39°43’54.00”W. Um dispositivo de GPS forneceu as informações de localização. Para garantir a identificação^(20)^, uma amostra representativa da espécie foi coletada e depositada no Herbário Dárdano de Andrade e Lima (parte da URCA) como exsicata (número de espécime 14208).

O óleo foi coletado perfurando-se aleatoriamente o tronco da árvore em dois locais diferentes, utilizando uma broca convencional com 2 centímetros (cm) de diâmetro e 45 cm de comprimento, com duas aberturas a 1 metro (m) e 1,50 m de altura. Após a drenagem da quantidade necessária de óleo, o furo foi selado com um tubo de PVC de 3/4 de polegada de diâmetro e 10 cm de comprimento, tampado com uma tampa de plástico. Para facilitar futuras coletas e evitar o acúmulo de resíduos de madeira, os tubos ou mangueiras podem ser removidos após a coleta, e fios de madeira podem ser fixados nos furos para impedir a entrada de pragas^(21)^. O material coletado foi armazenado em um recipiente opaco e mantido na geladeira para evitar a oxidação.

A formulação com 10% de OC foi obtida utilizando OM como diluente. O OM raramente causa reações alérgicas, não solidifica nem obstrui os poros e é utilizado para diluir óleos essenciais na prática farmacológica^(22)^.

### Análise dos resultados - efeito antioxidante

Ao final do período de tratamento, os animais foram dissecados para a preparação de um homogeneizado a 10% (p/v) em tampão fosfato 0,05 M a pH 7,4. Em seguida, os animais foram sacrificados para análise do efeito antioxidante.

A peroxidação lipídica foi analisada em amostras de homogeneizado de tecido obtidas da pele dos animais. As amostras foram adicionadas a um sistema catalítico de formação de radicais livres (FeSO4 0,01 mM e ácido ascórbico 0,1 mM) e mantidas a 37 °C por 30 minutos. A reação foi interrompida pela adição de ácido tricloroacético a 10%. Em seguida, as amostras foram centrifugadas a 3.000 rpm por 15 minutos. O sobrenadante foi removido e adicionado a ácido tiobarbitúrico a 0,8%. A mistura foi colocada em banho-maria por 15 minutos. Após o resfriamento, a absorbância foi medida a 535 nm. A peroxidação lipídica foi expressa em µmol de malondialdeído (MDA) por mg de tecido^(23)^.

A concentração de nitrito no homogeneizado de pele de rato foi determinada imediatamente após a dissecção de todos os grupos. Após centrifugação a 800×g por dez minutos, o sobrenadante foi coletado do homogeneizado, e a produção de óxido nítrico (ON) foi determinada com base na reação de Griess^(24)^. Para isso, 100 µL do sobrenadante foram incubados com 100 µL do reagente de Griess (sulfanilamida em 1% H₃PO₄/0,1% N-(1-naftil)etilenodiamina-dicloridrato/1% H₃PO₄/água destilada, 1:1:1:1) à temperatura ambiente por dez minutos. A absorbância foi medida a 550 nm usando um leitor de microplacas. Uma curva padrão foi criada usando várias concentrações de NaNO₂ variando de 0,75 a 100 µM, e os resultados foram expressos em µmol/g de proteína.

Para determinar a concentração de glutationa (GSH) reduzida, homogeneizados a 10% (p/v) em EDTA 0,02 M foram adicionados a uma solução de ácido tricloroacético a 50%. Após centrifugação a 3.000 rpm por 15 minutos, o sobrenadante homogeneizado foi coletado, e a concentração de GSH produzida foi determinada^(25)^. Em resumo, as amostras foram misturadas com tampão Tris-HCl de 0,4 M, pH a 8,9 e DTNB de 0,01 M. O nível de GSH foi determinado pela absorbância a 412 nm e expresso em ng/g de peso úmido do tecido de GSH.

### Análise estatística

Todas as análises foram realizadas utilizando análise de variância (ANOVA) com o *software* Prism 8.0.2 para *Windows* da *GraphPad Software* (San Diego, CA, EUA). Comparações múltiplas para significância foram conduzidas com ANOVA e testes post-hoc de Tukey. Os resultados são considerados significativos para p < 0,05 e apresentados como média ± Erro Padrão da Média^(26)^.

## RESULTADOS

Além disso, a administração de 10% de OC, AGE e OC na concentração de MDA em feridas de ratos diabéticos induzidas por aloxano e submetidas a diferentes regimes de administração durante um, sete ou 14 dias é evidente.

Em relação ao tratamento agudo com essas substâncias, não foram observados efeitos significativos nas concentrações de MDA quando comparados ao grupo SS (Figura 2A). No entanto, quando doses repetidas foram administradas ao longo de sete dias, verificou-se que tanto o OC (3,0 ± 0,6) quanto o AGE (8,4 ± 1,5) induziram uma redução significativa nas concentrações de MDA em comparação ao grupo SS (11,7 ± 0,9). Além disso, em comparação ao grupo OC, houve uma diminuição em relação aos grupos OM (10,5 ± 0,0), OC 10% (6,7 ± 1,6) e AGE (Figura 2B).

**Figura 2:** Efeitos do óleo de copaíba na concentração de malondialdeído em feridas de ratos diabéticos induzidos por aloxano. Cada barra representa a média ± Erro Padrão da Média dos grupos tratados com solução salina (SS), óleo mineral (OM), 10% de óleo de copaíba (OC 10%), ácido graxo essencial (AGE) e óleo de copaíba (OC)

*(A) Animais tratados por um dia; (B) Animais tratados com doses repetidas por sete dias; (C) Animais tratados com doses repetidas por 14 dias. Para todas as análises, p < 0,05 foi considerado significativo (análise de variância seguida pelo teste post-hoc de Tukey).*

No cenário de tratamento repetido ao longo de 14 dias, os grupos OM (12,0 ± 4,1), OC 10% (19,3 ± 1,6), AGE (9,8 ± 0,8) e OC (4,1 ± 0,6) demonstraram reduções consideráveis nas quantidades de MDA quando comparados ao grupo SS (35,2 ± 0,6). Além disso, essa diminuição foi igualmente notável nos resultados do grupo AGE em comparação com o grupo OC 10%, e no grupo OC em comparação com os grupos OM, OC 10% e AGE (Figura 2C).

### Determinação de nitrito

Foram construídas três representações gráficas para ilustrar os resultados da análise de nitrito, delineando os efeitos observados em relação a diferentes períodos de tratamento e substâncias aplicadas.

Na administração aguda do tratamento tópico, observou-se uma redução na concentração de nitrito ao usar OM (3,8 ± 0,6), OC 10% (3,2 ± 0,4), AGE (2,5 ± 0,4) e OC (2,9 ± 0,4) em comparação com SS (6,0 ± 0,4) (Figura 3A).

**Figura 3:** Efeitos do óleo de copaíba na concentração de nitrito em lesões de ratos diabéticos induzidos por aloxano. Cada barra representa a média ± Erro Padrão da Média dos grupos tratados com solução salina (SS), óleo mineral (OM), 10% de óleo de copaíba (OC 10%), ácido graxo essencial (AGE) e óleo de copaíba (OC)

*(A) Animais tratados por um dia; (B) Animais tratados com doses repetidas por sete dias; (C) Animais tratados com doses repetidas por 14 dias. Para todas as análises, p < 0,05 foi considerado significativo (análise de variância seguida pelo teste post-hoc de Tukey).*

Resultados semelhantes relacionados à concentração de nitrito, conforme descrito acima, foram evidentes nos grupos tratados com OM (3,3 ± 0,4), OC 10% (4,1 ± 0,2), AGE (2,3 ± 0,3) e OC (2,0 ± 0,1), por meio de doses repetidas durante sete dias, em comparação com SS (5,0 ± 0,3) (Figura 3B). Vale ressaltar que o OM apresentou uma concentração de nitrito menor em comparação com o grupo OC 10%, enquanto o AGE exibiu uma concentração menor quando comparado ao OM e ao OC 10%. Por outro lado, o tratamento com OC mostrou uma redução quando comparado ao OM, ao OC 10% e ao AGE.

Ao longo do tratamento repetido por 14 dias, tanto o grupo AGE (4,7 ± 0,1) quanto o grupo OC (1,3 ± 0,0) apresentaram uma diminuição na concentração de nitrito em comparação com o grupo SS (7,1 ± 1,0) (Figura 3C). O grupo AGE também apresentou uma redução quando comparado aos grupos OM (6,7 ± 0,4) e OC 10% (5,7 ± 0,4). Além disso, o tratamento com OC demonstrou maior significância na redução da concentração de nitrito em comparação aos grupos OM, OC 10% e AGE.

### Determinação da concentração de glutationa

Na fase aguda do tratamento, OM (577,7 ± 9,8), OC 10% (500,7 ± 7,6), AGE (472,7 ± 9,3) e OC (504,2 ± 0,0) apresentaram um aumento na concentração de GSH em comparação com SS (365,9 ± 10,2). Ao comparar os efeitos do tratamento com OM aos grupos OC 10%, AGE e OC após um dia de aplicação, verificou-se que o OM resultou em um aumento significativo na concentração de GSH (Figura 4A).

**Figura 4:** Efeitos do óleo de copaíba na concentração de glutationa em lesões cutâneas de ratos diabéticos induzidos por aloxano. Cada barra representa a média ± Erro Padrão da Média dos grupos tratados com solução salina (SS), óleo mineral (OM), 10% de óleo de copaíba (OC 10%), ácido graxo essencial (AGE) e óleo de copaíba (OC)

*(A) Animais tratados por um dia; (B) Animais tratados repetidamente por sete dias; (C) Animais tratados repetidamente por 14 dias. Para todas as análises, p < 0,05 foi considerado significativo (análise de variância seguida pelo teste post-hoc de Tukey).*

No tratamento com doses repetidas ao longo de sete dias, ficou evidente que os grupos AGE (2290 ± 169,1) e OC (986,7 ± 17,2) apresentaram um aumento na concentração de GSH em comparação com o grupo SS (414,8 ± 7,9) (Figura 4B). Além disso, ao longo desses sete dias, o tratamento com AGE apresentou um aumento maior quando comparado aos grupos que receberam OM (445,5 ± 69,8), OC 10% (552,6 ± 0,0) e OC. Da mesma forma, o grupo OC apresentou uma concentração de GSH maior em comparação aos grupos OM e OC 10%.

No contexto do tratamento com duração de 14 dias, destaca-se o desempenho dos grupos OM (571,7 ± 33,7), OC 10% (490,2 ± 22,8) e OC (1805,0 ± 34,9), todos apresentando um aumento significativo na concentração de GSH em comparação com o grupo SS (411,1 ± 9,1) (Figura 4C). Esses resultados positivos do tratamento com OC permaneceram consistentes quando comparados aos efeitos dos grupos OM, OC 10% e AGE (471,6 ± 9,5), mantendo concentrações elevadas de GSH. Além disso, o grupo OM aumentou a concentração de GSH em comparação com os grupos OC 10% e AGE.

## DISCUSSÃO

As análises demonstraram que o OC absoluto apresentou resultados superiores na redução da concentração de MDA em comparação com o AGE e os demais grupos de tratamento (SS, OM e OC 10%).

Neste contexto, a redução na concentração de MDA observada no grupo tratado com oleorresina absoluta de copaíba indica diminuição da peroxidação lipídica, um processo que pode ser crítico durante as fases inflamatória e proliferativa da cicatrização de feridas. O MDA é o produto final da peroxidação lipídica^(23)^, e sua redução sugere um controle eficaz do estresse oxidativo, o que pode favorecer a resolução da inflamação e a transição para a fase proliferativa. Isso poderia potencialmente reduzir os ataques oxidativos aos componentes celulares que são reparados durante o processo de cicatrização. Tais danos são frequentemente observados em ulcerações resultantes de complicações do DM sem tratamento antioxidante^(4,5)^. Resultados semelhantes^(18,27)^ demonstraram que a redução dos níveis de MDA está diretamente associada à melhora dos parâmetros inflamatórios e à aceleração da cicatrização de feridas em ratos diabéticos tratados com ácido alfa-lipóico, destacando a modulação do estresse oxidativo como um fator chave para a progressão adequada das fases de reparo tecidual.

Essa potencial atividade anti-inflamatória pode ser atribuída à presença de flavonoides e ácido caurenóico^(28)^ na oleorresina absoluta de copaíba, que contribuem para a eliminação de radicais livres e inibição da oxidação lipídica, prevenindo, assim, danos às membranas celulares^(29)^. Estudos^(30,31)^ indicam que esses compostos bioativos atuam modulando enzimas antioxidantes e diminuindo a atividade da mieloperoxidase, uma enzima envolvida na geração de *Reactive Oxygen Species* (ROS) durante a fase inflamatória.

Observou-se um aumento significativo na concentração de MDA no grupo SS no 14º dia, o que pode ser atribuído à progressão temporal do dano oxidativo às membranas celulares nas lesões de ratos diabéticos tratados com uma formulação sem agentes antioxidantes. O DM está associado ao aumento da peroxidação lipídica, resultando em níveis elevados de MDA, um marcador reconhecido de estresse oxidativo. Esse processo está ligado à hiperglicemia crônica característica do DM, que promove a produção excessiva de ROS, levando a danos oxidativos a lipídios, proteínas e DNA, juntamente com uma redução nas defesas antioxidantes endógenas^(32)^.

Em relação à modulação da concentração de nitrito, observou-se que, após sete dias de tratamento, apenas o AGE e o OC mantiveram atividade biológica efetiva na redução desse marcador, sugerindo que as demais formulações são mais eficazes durante a fase aguda do processo de reparo. O AGE apresentou maior atividade antioxidante em comparação com o OC 10% e o MO. Contudo, o OC exibiu desempenho superior na redução de nitrito, indicando um efeito mais sustentado e consistente na modulação do estresse oxidativo. Esses achados reforçam que a oleorresina de copaíba absoluta exerce um efeito terapêutico prolongado com capacidade de manter a homeostase redox ao longo do tempo.

Esse efeito pode estar associado à capacidade do OC de neutralizar ROS, particularmente o peróxido de hidrogênio (H₂O₂)^(33)^, o que está intimamente relacionado à sua eficácia na modulação do estresse oxidativo ao longo das diferentes fases da cicatrização de feridas no DM. Além disso, no contexto do DM, o H₂O₂ pode reagir com o NO para formar radicais peroxinitrito^(34)^, compostos altamente reativos que causam danos celulares significativos^(35)^. Esses danos podem prejudicar os estágios iniciais da cicatrização de feridas, como a fase inflamatória e a formação de tecido de granulação, atrasando, em última análise, o processo de reparo tecidual. Resultados semelhantes também foram relatados^(18,27)^, no qual descobriram que a redução do ON em feridas diabéticas leva a uma menor formação de radicais derivados de nitrogênio, promovendo redução da inflamação e melhor organização do tecido de granulação nas fases subsequentes de cicatrização.

Portanto, a aplicação tópica de OC pode representar uma abordagem terapêutica promissora para melhorar a cicatrização de feridas em indivíduos com DM, neutralizando as ROS e promovendo um ambiente redox propício à regeneração tecidual.

Outra enzima fundamental na defesa contra o estresse oxidativo é a GSH, um antioxidante endógeno que desempenha um papel crucial na manutenção da homeostase celular^(36)^. Este estudo observou que, durante a fase inflamatória inicial da cicatrização de feridas, particularmente comprometida em pacientes com DM devido ao elevado estresse oxidativo, houve um aumento nas concentrações de GSH em todos os grupos tratados. No entanto, o AGE apresentou um efeito máximo no sétimo dia, seguido por um declínio no décimo quarto dia, indicando que seu efeito antioxidante se limita aos estágios iniciais da cicatrização.

Em contraste, o OC demonstrou uma capacidade superior de manter as concentrações de GSH ao longo do tempo, inclusive durante a fase proliferativa e os estágios iniciais de remodelação tecidual, em comparação com outros tratamentos (SS, OM e OC 10%). Esse efeito sugere que o OC exerce um efeito antioxidante mais prolongado, o que é particularmente relevante no contexto do DM, em que a cicatrização de feridas é frequentemente prejudicada pelo estresse oxidativo persistente.

O efeito do OC pode ser parcialmente atribuído à presença do ácido caurenóico, cuja capacidade de estimular a síntese de GSH ou preservar seus níveis já foi demonstrada^(37)^. Este composto desempenha um papel fundamental no aumento das defesas antioxidantes endógenas, particularmente em condições hiperglicêmicas como as observadas no DM. Contribui para um microambiente bioquímico mais favorável à cicatrização de feridas, apoiando a progressão eficiente das fases de reparo. Resultados semelhantes^(18,27)^ indicam que níveis elevados de GSH são cruciais para acelerar a fase proliferativa e promover a deposição adequada da matriz extracelular e a reepitelização em feridas cutâneas diabéticas.

Por outro lado, o OM e o OC 10% não demonstraram eficácia significativa na manutenção dos níveis de GSH, apresentando respostas inferiores em comparação com o OC e o AGE. Este achado é corroborado por^(38)^, ao relatar que baixas concentrações de OC não exibem atividade antioxidante robusta, explicando a eficácia limitada observada na formulação a 10% utilizada neste estudo.

Com base nos resultados, conclui-se que a *Copaifera langsdorffii* Desf. demonstra um efeito antioxidante relevante no processo de cicatrização de feridas em animais com DM induzido, atuando na redução das concentrações de MDA e nitrito, enquanto promove um aumento nos níveis de GSH. O desempenho superior de OC, particularmente durante as fases inflamatória e proliferativa da cicatrização, destaca seu potencial terapêutico na mitigação do estresse oxidativo, um dos principais fatores que comprometem o reparo tecidual no DM. Contudo, pesquisas adicionais são necessárias para elucidar seus mecanismos moleculares e estabelecer protocolos clínicos padronizados que garantam sua aplicação segura e eficaz.

### Limitações do estudo

Esta pesquisa foi caracterizada como um estudo pré-clínico para elucidar o papel antioxidante da *Copaifera langsdorffii* Desf. na cicatrização de úlceras relacionadas ao diabetes. Estudos clínicos são necessários para incorporar antioxidantes como a *Copaifera langsdorffii* Desf. na prática clínica.

### Contribuições para área da saúde, enfermagem ou políticas públicas

A busca por terapias que minimizem o dano oxidativo em úlceras diabéticas tem sido frequente em unidades de tratamento de feridas. Nesse contexto, como contribuição para o avanço da prática assistencial e da ciência da enfermagem, este estudo apresenta resultados importantes de um recurso natural que demonstra evidências científicas, podendo ser utilizado na assistência de enfermagem para o tratamento de úlceras relacionadas ao diabetes.

## CONCLUSÕES

A oleorresina de copaíba demonstrou efeitos positivos na redução das concentrações de MDA após doses repetidas durante sete ou 14 dias de tratamento, diminuiu os níveis de nitrito em todos os períodos de tratamento e aumentou o potencial de GSH após doses repetidas durante sete e 14 dias.

Em conclusão, a oleorresina de *Copaifera langsdorffii* Desf. é um potencial agente terapêutico para o tratamento tópico dos parâmetros MDA, nitrito e GSH induzidos por aloxano em lesões de ratos.

## Data Availability

Os dados de pesquisa só estão disponíveis mediante solicitação.
